# Metagenomic Sequencing Reveals Distinct Gut Microbiome Profiles in Therapy-Naïve de Novo Pediatric Inflammatory Bowel Disease

**DOI:** 10.1093/ibd/izaf184

**Published:** 2025-09-12

**Authors:** Eva Vermeer, Femke M Prins, Iwan J Hidding, Jasmijn Z Jagt, Robert de Jonge, Marc A Benninga, Ranko Gacesa, Rinse K Weersma, Nanne K H de Boer, Tim G J de Meij

**Affiliations:** Department of Pediatric Gastroenterology and Hepatology, Emma Children’s Hospital, Amsterdam University Medical Centre, Amsterdam, The Netherlands; Amsterdam Gastroenterology Endocrinology Metabolism (AGEM) Research Institute, Amsterdam University Medical Centre, Amsterdam, The Netherlands; Amsterdam Reproduction & Development (AR&D) Research Institute, Amsterdam University Medical Centre, Amsterdam, The Netherlands; Department of Gastroenterology and Hepatology, University Medical Centre Groningen, Groningen, The Netherlands; Department of Cardiology, University Medical Centre Groningen, Groningen, The Netherlands; Department of Gastroenterology and Hepatology, University Medical Centre Groningen, Groningen, The Netherlands; Department of Pediatric Gastroenterology and Hepatology, Emma Children’s Hospital, Amsterdam University Medical Centre, Amsterdam, The Netherlands; Amsterdam Gastroenterology Endocrinology Metabolism (AGEM) Research Institute, Amsterdam University Medical Centre, Amsterdam, The Netherlands; Amsterdam Reproduction & Development (AR&D) Research Institute, Amsterdam University Medical Centre, Amsterdam, The Netherlands; Department of Laboratory Medicine, Amsterdam University Medical Centre, Amsterdam, The Netherlands; Department of Pediatric Gastroenterology and Hepatology, Emma Children’s Hospital, Amsterdam University Medical Centre, Amsterdam, The Netherlands; Amsterdam Gastroenterology Endocrinology Metabolism (AGEM) Research Institute, Amsterdam University Medical Centre, Amsterdam, The Netherlands; Amsterdam Reproduction & Development (AR&D) Research Institute, Amsterdam University Medical Centre, Amsterdam, The Netherlands; Department of Gastroenterology and Hepatology, University Medical Centre Groningen, Groningen, The Netherlands; Department of Genetics, University Medical Centre Groningen, Groningen, The Netherlands; Department of Gastroenterology and Hepatology, University Medical Centre Groningen, Groningen, The Netherlands; Department of Genetics, University Medical Centre Groningen, Groningen, The Netherlands; Amsterdam Gastroenterology Endocrinology Metabolism (AGEM) Research Institute, Amsterdam University Medical Centre, Amsterdam, The Netherlands; Department of Gastroenterology and Hepatology, Amsterdam University Medical Centre, Amsterdam, The Netherlands; Department of Pediatric Gastroenterology and Hepatology, Emma Children’s Hospital, Amsterdam University Medical Centre, Amsterdam, The Netherlands; Amsterdam Gastroenterology Endocrinology Metabolism (AGEM) Research Institute, Amsterdam University Medical Centre, Amsterdam, The Netherlands; Amsterdam Reproduction & Development (AR&D) Research Institute, Amsterdam University Medical Centre, Amsterdam, The Netherlands

**Keywords:** pediatric inflammatory bowel disease, Crohn’s disease, ulcerative colitis, gut microbiome, shotgun metagenomics

## Abstract

**Background and Aims:**

Microbiome studies reveal distinct microbial differences in inflammatory bowel disease (IBD), indicating its potential role in pathophysiology and as a noninvasive diagnostic biomarker. This study aims to profile the gut microbiome in children with IBD, compared to both healthy controls (HC) and controls with gastrointestinal symptoms (CGI), and to assess the potential of microbiome profiles as noninvasive diagnostic markers for de novo treatment-naïve pediatric IBD, and as early predictive markers for therapy response.

**Methods:**

We analyzed baseline fecal samples and clinical data from 103 therapy-naïve children with IBD, 75 CGI, and 356 age and sex matched HC. Metagenomic sequencing was performed, and prediction models assessed diagnostic potential and prediction of induction therapy response at 3 months.

**Results:**

Alpha diversity progressively decreased from HC to CGI (*P *< .001) and decreased even further in IBD patients (*P *= .0056). Beta diversity analysis showed significant clustering differences (*P *< .001, R^2^ = 0.045). Differential abundance analysis revealed 116 species differing between HC and IBD, and 30 species between CGI and IBD. Prediction models based on microbiome features accurately distinguished IBD from HC (area under the curve [AUC] = 0.96) and from CGI (AUC = 0.71). However, these models were outperformed by clinical features, such as fecal calprotectin. Microbiome-based prediction of response to induction therapy in general showed limited accuracy (AUC = 0.63), as well as for response to nutritional induction therapy (AUC = 0.67).

**Conclusions:**

We observed profound gut microbiome differences between de novo, therapy-naïve pediatric IBD patients and controls. While microbiome profiles hold promise for improving diagnostic precision, their predictive value for therapy response seems limited.

Key messages *What is already known?*

Gut microbiome alterations have been linked to pediatric inflammatory bowel disease (IBD), and microbial profiles differ between patients and healthy individuals. However, the diagnostic and predictive utility of these profiles remains uncertain.

*What is new here?*

This study demonstrates that gut microbiome profiling can accurately distinguish children with from both healthy controls (HC) and controls with gastrointestinal symptoms (CGI), with diagnostic models achieving AUCs of 0.96 and 0.71, respectively. Notably, the distinction between therapy-naïve IBD and CGI—often a diagnostic challenge in clinical practice—has been rarely explored and is a key strength of this work.

*How can this study help patient care?*

Microbiome-based diagnostics may aid in the noninvasive detection of pediatric IBD, potentially reducing the need for endoscopy—particularly in cases where symptoms and clinical features are inconclusive. While not yet predictive of treatment response, microbiome profiling offers promising avenues for earlier diagnosis and personalized care strategies.

## Introduction

Inflammatory bowel disease (IBD), including Crohn’s disease (CD), ulcerative colitis (UC), and IBD-unspecified (IBD-U), is a chronic condition characterized by recurring intestinal inflammation. In recent decades, there has been an upward trend in the incidence of IBD, accompanied by a decrease in the age at which patients present, with approximately 25% of cases currently initiating before the age of 20.^1–3^

Typical clinical symptoms of IBD include abdominal pain, diarrhea, and rectal blood loss, but children also may present with atypical symptoms, such as fatigue, weight loss, growth failure, delayed puberty, or extraintestinal manifestations.^4^ Moreover, the currently available biomarker fecal calprotectin (FCP) is highly sensitive for detecting inflammation but is limited by a specificity of 68%.^5^ Hence, IBD diagnosis is often delayed especially in the pediatric population, while children are at a greater risk of complications and more severe disease course.^6^^,^^7^ Endoscopy remains the gold standard for diagnosing IBD.^8^ This is a relatively costly and invasive procedure, and children often require hospital admission for bowel cleansing via a nasogastric tube and undergo the procedure under general anesthesia. Novel noninvasive biomarkers are therefore needed for early detection of disease, timely treatment initiation, minimizing risk of complications, and preventing unnecessary endoscopies.

IBD is viewed as a multifactorial disease, resulting from complex pathogenetic mechanisms to which the intestinal microbiome, host genetics, immune responses, and environmental factors all contribute.^9^ In recent years, the role of the gut microbiome in adult IBD has been intensively studied. It is characterized by dysbiosis with a decrease in alpha diversity, an increase in facultative anaerobes such as *Escherichia coli*, and a decrease in obligate anaerobes such as *Fecalibacterium prausnitzii,* and *Roseburia* and *Alistipes* species.^10^ Evidence suggests that pediatric IBD has a stronger genetic component than adult-onset IBD and may, therefore, represent a distinct disease entity with unique microbial changes.^11^ The exact underlying pathogenesis and whether altered intestinal microbial composition is either a cause or a consequence of inflammation in IBD remains largely unknown, but it does provide opportunities for the development of novel diagnostic biomarkers and therapeutic interventions.^12^

Nutritional therapy plays a significant role in the management of pediatric CD and is hypothesized to act through modulation of the gut microbiome.^13^^,^^14^ Maintaining adherence to these strict dietary rules is challenging, with an approximately 25%–35% rate of noncompliance, necessitating escalation to pharmacological treatment such as thiopurines or even antitumor necrosis factor (TNF) therapy.^15^^,^^16^ Due to the large heterogeneity in IBD, both in disease localization and severity, and its complex pathogenesis, it is currently challenging to predict at baseline which patients will benefit from nutritional therapy and from a conventional step-up treatment approach, and which patients would benefit from a more aggressive form of induction treatment to improve clinical outcome and reduce the risk of complications.^17^ Microbiome profiling at diagnosis may offer a solution by enabling personalized induction treatment regimens, predicting individual responses, minimizing unnecessary drug exposure, and optimizing clinical outcomes.^18^^,^^19^

The aim of this study was to profile the gut microbiome of de novo, therapy-naïve pediatric IBD patients, controls with gastrointestinal symptoms (CGIs), and healthy controls (HCs) using metagenomic sequencing and to evaluate the potential of the gut microbiome as a noninvasive diagnostic tool for pediatric IBD detection. A secondary aim was to determine whether baseline microbial profiles can serve as predictive markers for response to induction therapy in general, and response to nutrition-based induction treatment in particular.

## Materials and Methods

### Study population

In our study, we combined 2 different cohorts from The Netherlands, of which extensive demographic and clinical data were available, as well as fecal samples. The Rapid cohort is a prospective hospital-based cohort of therapy-naïve children with gastrointestinal symptoms, some of whom were diagnosed with IBD or another gastrointestinal disease. The LifeLines cohort is a large population-based cohort from the Dutch Microbiome Project, from which we selected healthy children without any known disease and medication use. We matched participants from the LifeLines cohort to patients in the Rapid cohort based on age and sex. This matching was performed at a ratio of 2:1 using the *MatchIt* package (v4.5.5, https://cran.r-project.org/web/packages/MatchIt/index.html) with the “nearest” method. Further details on both cohorts are described below.

#### Rapid cohort

Between December 2017 and June 2023, patients were included in the Rapid cohort if they were aged between 4 and 18 years, referred to the outpatient clinics of the Department of Pediatric Gastroenterology of the Amsterdam University Medical Centre (both locations AMC and VUmc), had suspected IBD or disorders of gut-brain interaction (DGBI) such as functional abdominal pain-not otherwise specified (FAP-NOS) or irritable bowel syndrome (IBS). Patients who had used immunosuppressants prior to inclusion, taken antibiotics or probiotics in the 3 months before baseline, or had an immunocompromising disease were excluded from the cohort.

Patients referred under suspicion of IBD underwent the diagnostic work-up according to current guidelines, including clinical assessment, laboratory tests, radiology, and endoscopy with histology, if indicated.^8^ When IBD diagnosis was confirmed, patients were placed in the IBD group, and when IBD was ruled out, patients were allocated to the CGI group. Patients referred under suspicion of a DGBI followed the indicated diagnostic route according to the Rome IV criteria,^20^ including a fecal calprotectin (FCP) test to rule out inflammation, and were directly allocated to the CGI group.

All patients were required to collect a stool sample in a prospective study design, before bowel cleansing if applicable, in a provided sterile container. They were instructed to store the sample in their home freezers within 1 hour after collection and bring it cooled to their next hospital visit. The storage duration in home freezers did not exceed a couple of weeks. Subsequently, the sample was stored at −80°C in the hospital until further handling. This standardized method of stool collection aligns with previously published work on the fecal microbiome, where we have not observed inconsistencies in sample integrity or analysis.^21^^,^^22^

Demographic and clinical data were collected at baseline from the electronic patient records and included sex, age, height, weight, body mass index (BMI), FCP, C-reactive protein (CRP), and Pediatric Crohn’s Disease Activity Index (PCDAI) or Pediatric Ulcerative Colitis Activity Index (PUCAI) score, if applicable. In the IBD group, patients were followed up at 3 months after initiation of induction therapy. Additional clinical data were then collected regarding response to induction therapy and disease activity, including FCP, CRP, and PCDAI or PUCAI score. Response was defined as both clinical response, marked by a reduction of ≥12 points in PCDAI in case of CD or a reduction of ≥20 points in PUCAI in case of UC or IBD-U, and absence of the need for treatment escalation to corticosteroids or biological agents within the initial 3 months of therapy.

#### LifeLines cohort

The LifeLines cohort is a previously described 3-generational population cohort from the northern Netherlands, characterized by comprehensive data collection, including stool sample collection for the Dutch Microbiome Project.^21^ Similar to RAPID, all participants were asked to collect a stool sample at home and freeze this within 15 minutes. This was then later collected by lifelines personnel and stored at −80°C until further handling. We selected and matched participants from the LifeLines cohort as described previously and included them in this study as HCs (https://ega-archive.org/studies/EGAS00001005027).

### DNA extraction, sequencing, and metagenomic data processing

The method for preparing and processing metagenomic sequencing data of the Rapid cohort was consistent with earlier studies within the LifeLines cohort, as described previously.^21^ Microbial DNA was extracted from fecal samples using the QIAamp Fast DNA Stool Mini Kit (Qiagen) and the QIAcube (Qiagen) automated sample preparation system, following the manufacturer’s instructions. Metagenomic sequencing was performed at NovoGene Cambridge using the Illumina NovaSeq 6000 S4 flowcell with PE150 to generate reads for each sample. The metagenomic reads, provided in *fastq* format, underwent trimming using KneadData tools. This involved filtering out reads below Phred quality score 30 and removing Illumina adapters. Reads that mapped to the human genome were removed with the Bowtie2 tool. As a final quality control step, samples with a total read depth <1 million (*n* = 6) were excluded from further analysis. For taxonomy assignment, we used the MetaPhlan4 tool with the database of marker genes (version October 2022). We profiled genes encoding microbial metabolic pathways using the HUMAnN 3.6 pipeline to also study the functional capabilities of the microbiome. In total, we detected 4218 bacterial taxa and 480 pathways across the quality-controlled Rapid dataset. The metagenomic data from the LifeLines samples were processed in the same way, with the same database of marker genes, resulting in 5066 bacterial taxa and 483 pathways. The data from both cohorts were combined for the species and pathway levels, with missing species and pathways in one cohort but present in the other cohort imputed with zeros. To deal with sparse microbial data in our analyses, we applied filtering. For each group (HC, CGI, and IBD cases) we selected bacterial species with a mean relative abundance of >0.01% and pathways with a mean relative abundance of >0.0001%, which were present in at least 20% of participants in the respective group; we removed the unknown fractions. This resulted in 212 filtered species and 132 filtered pathways in the HC group, 184 species and 152 pathways in the CGI group, and 146 species and 161 pathways in the IBD group. These species and pathways were used for analyses. Depending on the comparison tested, we included the features from the relevant groups and rescaled them before testing.

### Microbiome analyses

We examined the diversity and dissimilarity in gut microbiome composition across various groups of interest, including IBD cases vs CGI cases vs HC, and responders vs nonresponders. To investigate microbial richness (number of microbes) and evenness (abundance patterns of these microbes) within samples (alpha diversity), we calculated the Shannon diversity index at species level using the *diversity* function in the R package *vegan* (version 2.6-4, https://cran.r-project.org/web/packages/vegan/index.html). The diversity index was calculated on the nonfiltered dataset. For pathways, we calculated the richness because it is more sensitive to rare features. Multivariable linear regression was used to test differences in diversity index between groups, adjusting for age, sex, and read depth. Because of the compositional nature of metagenomic data, we applied a centered log-ratio (CLR) transformation to the data for downstream analyses. Zeros were replaced with a pseudocount equal to half of the minimum relative abundance in the dataset. Microbial and functional dissimilarity between samples (beta diversity) was determined by calculating Aitchison distances using the *vegdist* function. Principal coordinates analysis (PCoA) plots were generated to visualize group similarities and differences. To determine the proportion of explained variance of phenotypes on beta diversity, we used permutational multivariable analysis of variance (PERMANOVA) implemented in the *adonis2* function with 1000 permutations, adjusting for age and sex. For differential abundance analysis, we used linear models using the *lm* function from base R (version 4.2.3, https://www.rdocumentation.org/packages/stats/versions/3.6.2), adding sex and age as covariates. We calculated the estimated marginal means for each group using the *emmeans* package (v1.8.9, https://cran.r-project.org/web/packages/emmeans/index.html). Multiple testing in the differential abundance analysis was accounted for by using the Benjami-Hochberg correction, with a false discovery rate (FDR) of <0.05 considered statistically significant. An a priori power analysis was not performed, as standard methods for such analysis are not well-established for complex microbiome data.

### Microbiome modeling and predictions

To model the microbiome for evaluating diagnostic potential and perform prediction analyses for therapy response, we used the R package *Coda4Microbiome* (version 0.2.3, https://cran.r-project.org/web/packages/coda4microbiome/index.html). The dataset was randomly split into a 75% training set and 25% test set. In the training set, we modeled the ratio between species or pathways using elastic net regularized generalized linear regression, implemented in the *coda_glnet* function. This method was used to identify the microbial signatures that best explain differences between the groups of interest. We used 10-fold cross validation if the number of cases per group was larger than 10, otherwise 5-fold cross validation was applied. To prevent overfitting, the maximum number of features (*nvar*) setting was set at 15, to force selection of smaller, more interpretable ratios. In the test set, the identified species or pathway ratios were calculated for each sample. To assess the predictive value of each data layer, we constructed separate linear models for species, pathways, and clinical features (age, sex, and if available FCP and CRP). Additionally, combined models incorporating all data layers were created to evaluate the predictive power. Subsequently, receiver operating characteristic-area under the curve (ROC-AUC) values were computed for each model. To properly account for variability in the data and ensure robust estimates, we repeated the train-test split process with 50 permutations. The average AUC values and their standard deviations across permutations were plotted to reflect the overall predictive accuracy and variability. While these permutations allow for a reliable estimation of the predictive power, this introduces potential variation in the features selected across permutations.^23^ Therefore, to identify the most stable predictors, we applied the *coda_glmnet* function to the whole dataset and used these features for interpretation. We also tested other prediction algorithms, using the *svm* function from the e1071 (version 1.7-16, https://cran.r-project.org/web/packages/e1071/index.html) R package for support vector machine (SVM) prediction, the *cv.glmet* function from the *glmnet* (version 4.1-8, https://cran.r-project.org/web/packages/glmnet/index.html) package for Least Absolute Shrinkage and Selection Operator (LASSO) prediction and the *randomForest* (version 4.7-1.2, https://cran.r-project.org/web/packages/randomForest/index.html) function from the *randomForest* R package. All with default settings.

## Results

### Study population

From the Rapid cohort, we included a total of 178 subjects, 103 of whom were diagnosed with IBD (70 CD, 27 UC, 6 IBD-U), and 75 of whom were allocated to the CGI group. Of all 178 individuals, 134 were initially referred under suspicion of IBD (75.3%), and 44 were initially referred under suspicion of DGBI (24.7%) and placed in the CGI group. Of those 134 with IBD suspicion, a small portion (5.2%) ultimately did not undergo an endoscopy because the IBD diagnosis was already ruled out in an earlier stage of the diagnostic work-up. Two individuals in the CGI group were referred under suspicion of a juvenile polyp and underwent an endoscopy for polypectomy. For the LifeLines cohort, we used 356 matched individuals for the HC group. The baseline characteristics of the study population are listed in Table 1. There were no significant differences in sex or BMI between the study groups, but age was statistically significantly lower in the HC group (*P *= .018). The FCP and CRP levels were significantly higher in the IBD group than in CGI (both *P *< .001). The definitive diagnoses of the CGI group are displayed in Table S1; almost half (45.3%) of the CGI group was diagnosed with IBS. No significant differences were found in IBD phenotypes when comparing sex, age, and BMI (Table S2**)**. Data to evaluate treatment response 3 months after initiation of induction therapy were available for 67 out of the 103 IBD patients (42 CD, 19 UC, 6 IBDU). Patients who received steroids or an anti-TNF agent as induction therapy were excluded from response classification (*n* = 15), and clinical scores or therapy data at either baseline or at 3 months were missing for an additional 21 patients. Among those with available data, 30 were classified as responders and 37 as nonresponders. There were no significant differences in age, sex, BMI, PCDAI/PUCAI at baseline, FCP, CRP, or IBD phenotypes between both response groups at baseline (Table S3**)**.

**Table 1.** Patient characteristics of the study population, *n* (%) or median (IQR).

	Healthy controls (HC)	Controls with gastrointestinal symptoms (CGI)	Inflammatory bowel disease (IBD)	***P* value** ^b^
	*n* = 356	*n* = 75	*n* = 103	
Female	163 (46%)	41 (55%)	54 (52%)	0.244
Age, years	13 (11-15)	14 (11.5-16)	15 (13-16)	**0.018**
Body mass index^a^, kg/m^2^	19 (17.3-20.8)	19.1 (17-21.5)	18.9 (16.2-21.4)	0.536
Fecal calprotectin^a^, µg/kg	NA	26 (11-280)	1963 (1074-3219)	**<0.001**
CRP^1^, mg/L	NA	1 (0-3)	8 (1-28)	**<0.001**
Reads, million	14.1 (13-15.2)	12.5 (11.4-14.1)	12.0 (9.0-13.5)	**<0.001**

a Missing values in one or more of the groups.

b Pearson χ^2^ test for categorical variables, Kruskal-Wallis test for numerical variables with 3 groups, and Mann-Whitney *U* test for numerical variables with 2 groups. P < 0.05 was considered statistically significant.

### Gut microbiome diversity and composition across HC, CGI, and IBD groups

**Figure 1.**
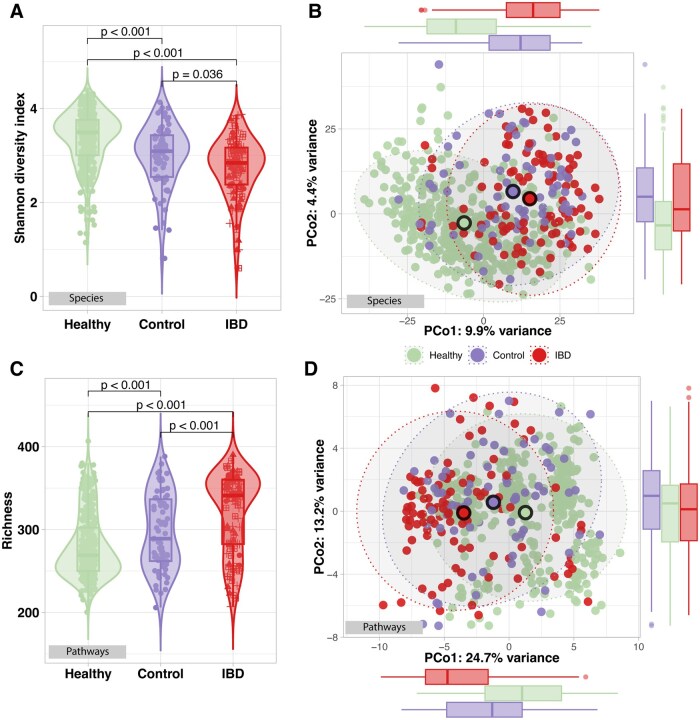
Alpha and beta diversity between samples of healthy controls, controls with gastrointestinal complaints and IBD cases at species and functional level. **(A)** Shannon diversity at species level of the different groups. *P* values indicate the significance tested in a linear model including age, sex, and reads as covariates. **(B)** Aitchison distance at species level. Dots represent individual samples, colored by group. **(C)** Richness of functional pathways for all groups. *P* values indicate the significance tested in a linear model including age, sex, and reads as covariates. **(D)** Aitchison distance for pathways. Dots represent individual samples, colored by group.

We investigated whether gut microbiome diversity differed across HC, CGI, and the IBD group by assessing alpha diversity using the Shannon index. Diversity progressively decreased from HC (mean = 3.36, SE 0.03) to CGI (mean = 3.00, SE 0.08), and even further in IBD patients (mean = 2.74, SE 0.06) (Figure 1A, all comparisons *P *< .05). When stratifying the IBD group by IBD phenotype and comparing it to CGI, we found a significant reduction in Shannon diversity in CD compared to CGI (*P *= .023) but no significant difference between UC and CGI. Similarly, within the CGI group we compared different phenotypes to HC and found that Shannon diversity was significantly lower in IBS compared to HC (*P *< .001), while no significant difference was observed between FAP-NOS and HC. Comparing IBD-phenotypes (CD vs UC) did not show differences in Shannon diversity between phenotypes (*P *= .66, Figure S1A).

Next, we assessed beta diversity by visualizing the Aitchison distance between samples using PCoA. Clear clustering was observed between the groups, particularly separating HC from both CGI and IBD groups (Figure 1B), where the separation between CGI and IBD was more limited. PERMANOVA analysis, adjusted for age and sex, confirmed that group (ie, IBD, CGI, HC) accounted for a significant part of the variance in microbial composition, although with a small effect size (R^2^ = 0.045, *P *< .001), demonstrating that the gut microbiome in IBD patients is distinct from both CGI and HC, as well as CGI differing significantly from HC.

**Figure 2.**
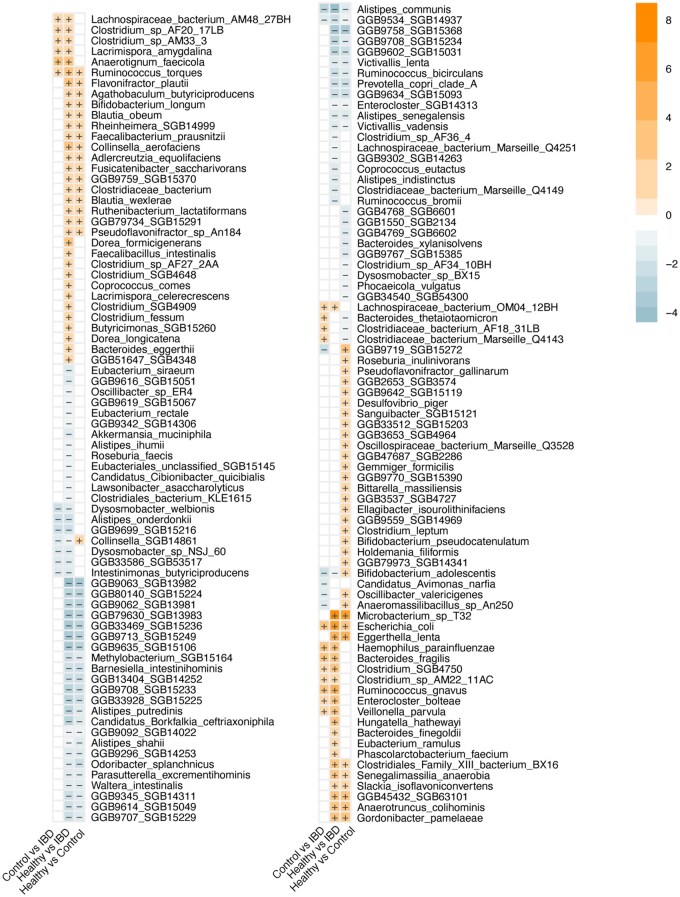
Differential abundance of species between healthy controls, controls with gastrointestinal complaints and IBD cases. The color and sign in the boxes indicate the value and direction of the coefficient. All FDR significant findings are shown. The first named group is the reference group for the linear model.

We then investigated which species were differentially abundant across the groups (Figure 2). A total of 117 species showed significant differences between HC and IBD patients, including a notable increase *in F. prausnitzii* (estimate 1.99, FDR < 0.001) and *Escherichia coli* (estimate 6.06, FDR < 0.001) in IBD. In the comparison between HC and CGI, 97 species were differentially abundant, with an increase in *Eggerthella lenta* (estimate 4.09, FDR < 0.001) in CGI. When comparing CGI and IBD, 32 species were differentially abundant, including a decrease in *Alistipes communis* (estimate −2.62, FDR = 0.004) and an increase in *E. coli* (estimate 3.41, FDR < 0.001) in IBD. When comparing IBD phenotypes, we did not find differential abundant species between UC and CD.

Regarding the functional potential of the gut microbiome, we observed a significant increase in pathway richness in IBD compared to both HC and CGI (*P *< .001 for both comparisons) (Figure 1C). Beta diversity analysis also revealed distinct clustering patterns among HC, CGI, and IBD, indicating differences in functional potential of the microbiome across groups (*P *< .001, R^2^ = 0.066) (Figure 1D). These differences were confirmed by differential abundance analysis, which identified 127 differentially significant abundant pathways between HC and IBD, 72 pathways between HC and CGI, and 42 pathways between CGI and IBD. In HC vs IBD, adenosine and guanosine nucleotide degradation pathways, as well as palmitate biosynthesis, were significantly increased in IBD (estimate 0.91, 0.76, and 2.91, respectively), and a pathway involved in folate transformation was reduced in IBD (estimate -0.30). For HC vs CGI, we found that CGI patients showed a significant decrease in pyramidine ribonucleotide synthesis (estimate -0.25) and a decrease in flavin biosynthesis (estimate -0.19). In the comparison between CGI and IBD, IBD patients showed again increased adenosine nucleotide degradation (estimate 0.50) and palmitate biosynthesis (estimate 1.42) and a decrease in 5-aminoimidazole ribonucleotide biosynthesis (estimate -0.16). Comparing CD with UC within the IBD group revealed no FDR significantly different abundant pathways. All significantly different pathways between the listed comparisons are visualized (Figure S2).

### Gut microbiome as diagnostic marker for pediatric IBD

**Figure 3.**
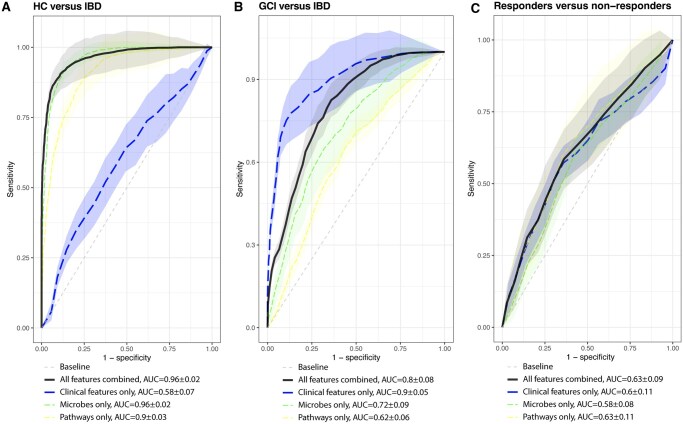
AUC curves of microbiome predictions. **(A)** This shows the predictive power of clinical features, microbial features, and pathway features, and all features combined for distinguishing HC from IBD. **(B)** Predictive power for distinguishing CGI vs IBD. **(C)** AUC curve for prediction of response to induction therapy.

We aimed to classify individuals as HC, CGI, or IBD based on their microbiome signatures, using the *Coda4Microbiome* package to select bacterial species and pathways.^24^ Linear models were applied to assess the predictive power of these microbiome features. Since FCP and CRP measurements were unavailable for HC, we used only age and sex as clinical features in these prediction models. The models distinguishing HC from IBD showed an AUC of 0.56 ± 0.07 for clinical features, 0.96 ± 0.02 for species, and 0.89 ± 0.03 for pathways. When combining all layers, the model achieved an AUC of 0.96 ± 0.02 (Figure 3A), mainly driven by microbial species, particularly increased abundance of *E. coli* (model coef = 0.26) and decreased abundance of *A. communis* (model coef = -0.25) in the IBD group. Notably, the increased abundance of *F. prausnitzii* (model coef 0.07) in the IBD group was also a key distinguishing feature for IBD, as well as the increased abundance of the adenosine nucleotide degradation pathway (model coef 0.21).

In distinguishing HC from CGI, the AUC was 0.56 ± 0.07 for clinical features, 0.94 ± 0.03 for species, and 0.84 ± 0.07 pathways. The combined model resulted in an AUC of 0.94 ± 0.03, again primarily driven by microbial species. Specifically, the increased abundance of *Odoribacter splanchnicus* (model coef = 0.53) and decreased abundance of *Senegalimassilia anaerobia* (model coef = -0.29) were strong predictors for identifying HC compared to CGI. For pathways, a strong predictor was the abundance of 5-aminoimidazole ribonucleotide biosynthesis (model coef -0.25) for HC.

For distinguishing CGI from IBD, where FCP and CRP measurements were available, the clinical features model demonstrated the strongest performance with an AUC of 0.90 ± 0.05, while species and pathways achieved AUCs of 0.71 ± 0.07 and 0.61 ± 0.08, respectively. When all layers were combined, the AUC reached 0.80 ± 0.06 (Figure 3B), suggesting that clinical features alone provide the best classification between CGI and IBD, and the addition of microbiome features did not enhance the model. The species model was mainly driven by increased abundance of *E. coli* and *Anaerotignum faecicola* in the IBD group, and increased abundance of *GGB9719_SGB15272* (part of the *Oscillospiraceae* family) as the primary feature for CGI. For the pathway model, 4-deoxy-L-threo-hex-4-enopyranuronate degradation (model coef 0.31) abundance was a strong predictor for IBD. As this was our worst species-based prediction, we compared different prediction models. LASSO and SVM models achieved similar or worse predictions (AUC = 0.70 ± 0.08 and AUC = 0.68 ± 0.07). Random forest modeling resulted in an improved prediction with an AUC of 0.81 ± 0.06 based on species alone, indicating a potential effect of the abundance of single species rather than a ratio between multiple species. This prediction was primarily based on the abundances of species *Lawsonibacter asaccharolyticus*, *A. faecicola*, and *E. coli* (MeanDecreaseGini > 1.4).

To analyze microbial differences between CD and UC in our cohort, we ran the same prediction models as before. We observed a split based on microbial features with an AUC of 0.74 ± 0.11. Clinical features alone resulted in an improved AUC of 0.78 ± 0.11 due to the difference in CRP as indicated by Table S2. Pathways showed limited predictability with an AUC of 0.63 ± 0.13. When microbial features, clinical features, and pathways were all combined, this resulted in an AUC of 0.76 ± 0.10.

Using the subspecies-level annotation from MetaPhlan4’s single genome bins (SGBs), we identified which species in our dataset contained multiple SGBs. Focusing on the species identified as predictive in our models, we detected 8 SGBs of *F. prausnitizii* and 2 SGBs of *Ruminococcus torques*. In the model distinguishing HC from IBD, we found that the increased abundance of *F. prausnitizii SGB15316* (model coef = 0.06) was predictive for IBD, while the other 7 SGBs did not contribute. For *R. torques*, we found that the increased abundance of *SGB4608* was highly predictive for IBD (model coef = 0.21).

### Gut microbiome and prediction of therapy response

We investigated the potential of the gut microbiome to predict response to all types of induction therapy in pediatric IBD patients. We compared microbial diversity and composition between responders (*n* = 30) and nonresponders (*n* = 37) at baseline and found no significant difference in Shannon diversity between the 2 groups (*P *= .92). Similarly, beta diversity analysis showed no significant differences in microbial composition (*P *= .78), and no species were identified as differentially abundant between the groups. Comparing the functional profiles of the gut microbiome between the groups also did not show differences between responders and nonresponders at baseline. When using microbial features to predict induction therapy response, the model showed an AUC of 0.58 ± 0.08 for species and 0.63 ± 0.11 for pathways. Combining microbial and the clinical features (CRP, FCP, age, and sex) resulted in an AUC of 0.63 ± 0.09, which had a marginally better AUC but a significantly better model fit (χ^2^  *P *= 8.94e-15) than the clinical model alone (AUC = 0.60 ± 0.11) (Figure 3C).

Next, we focused specifically on the subgroup of patients that started with nutritional induction therapy, both exclusive enteral nutrition (EEN) or Crohn’s disease exclusion diet (CDED), as these therapies are hypothesized to act through modulation of the gut microbiome. Comparisons of Shannon diversity between responders to nutritional therapy (*n* = 17) and nonresponders to nutritional therapy (*n* = 23) again revealed no significant differences at baseline (*P *= .90), and microbial composition (beta diversity) was similar between the groups. Predictive models using microbial species had an AUC of 0.65 ± 0.13, while the model using pathways yielded an AUC of 0.64 ± 0.12, indicating no strong predictive capacity of microbial features for response to nutritional induction therapy. However, including microbial features in addition to the clinical model (AUC 0.67 ± 0.13) resulted in the highest AUC, and the clinical model alone (0.63 ± 0.15) did not perform better than the separate microbiome features.

### Post hoc analysis on gut microbiome to prevent unnecessary endoscopies

Our prediction analysis showed that clinical features, including inflammatory markers (FCP, CRP) effectively distinguish IBD from CGI (AUC 0.90 ± 0.05). However, some CGI cases still were clinically suspected of IBD, leading to invasive and costly endoscopic procedures. This prompted us to perform post hoc analyses to explore baseline gut microbiome differences between patients who underwent, in hindsight, an unnecessary endoscopy (*n *= 19) and those who underwent necessary endoscopy (*n* = 109). An unnecessary endoscopy was defined as an endoscopy performed in a control patient who, based on clinical features, was suspected of having IBD but was found not to have IBD following the procedure. Of the patients, 17 had elevated FCP (>50 µg/kg) and 5 had elevated CRP (>5 mg/g) at baseline. A necessary endoscopy was defined as an endoscopy performed in a patient who was suspected of having IBD and was subsequently confirmed to have IBD, or in a patient suspected of having a condition for which endoscopy is also an appropriate diagnostic tool, such as celiac disease or polyps. Our goal was to determine whether microbiome features could serve as biomarkers to reduce unnecessary endoscopies.

Alpha diversity was lower in the necessary endoscopy group (mean 2.76, SE 0.06) compared to the unnecessary endoscopy group (mean 3.08, SE 0.12), with the difference nearing statistical significance (*P *= .076). PERMANOVA analysis revealed a significant difference in beta diversity between the 2 groups (*P *= .002, R^2^ = 0.014). Additionally, *E. coli*, *Lacrimispora amygdalina* and *Clostridium spAF2017LB* abundance was significantly decreased in the unnecessary endoscopy group compared to the necessary endoscopy group. Functionally, richness was higher in the necessary endoscopy group (*P *= .01), and beta diversity also differed between groups (*P *= .01, R^2^ = 0.02). Two pathways related to nucleotide degradation were decreased in the unnecessary endoscopy group, while a pathway involved in N-acetyl-D-glucosamine degradation was increased in the group with unnecessary endoscopies.

To evaluate the predictive power of microbial features in distinguishing necessary from unnecessary endoscopies, we modeled microbial species and pathways. The clinical features model yielded an AUC of 0.76 ± 0.11, microbial species achieved an AUC of 0.67 ± 0.11, and pathways reached an AUC of 0.71 ± 0.11. Combining these layers resulted in an AUC of 0.69 ± 0.1. The microbial pathways involved in flavin and Coenzyme A biosynthesis were predictive of needing an endoscopy, while pathways for pyrimidine deoxyribonucleosides salvage and chorismate biosynthesis were associated with the unnecessary endoscopy group. Random forest models achieved a marginally improved AUC of 0.73 ± 0.11, primarily based on the abundance of *Bifidobacterium adolescentis.*

## Discussion

Our study aimed to profile the gut microbiome of de novo, therapy-naïve pediatric IBD patients, CGI, and HC and to describe the differences between these 3 groups. Additionally, it sought to assess whether the microbial profiles at diagnosis could be used as diagnostic markers for pediatric IBD and predict response to induction treatment in general as well as nutritional therapy. Our study demonstrates distinct gut microbiome compositions between HC, CGI, and pediatric IBD cases. Our diagnostic model including microbiome features achieved an impressive AUC of 0.96 for distinguishing between HC and IBD, and an AUC of 0.80 for differentiating CGI from IBD. However, the ability of microbiome-based models to predict response to induction therapy in general showed limited accuracy (AUC 0.63), and an AUC of 0.67 for response to nutritional induction therapy.

### Gut microbiome composition

To our knowledge, this is the first study to perform gut microbiome analyses through metagenomic sequencing in a pediatric cohort of de novo therapy-naïve IBD patients, CGI, and HCs. In the past decade, several studies have been conducted on the gut microbiome in pediatric IBD patients. All but one study compared the gut microbiome of pediatric IBD patients to that of HCs.^25–32^ Kellermayer et al compared gut microbial compositions of pediatric IBD patients with those of children in whom IBD diagnosis was excluded following endoscopic and histologic analysis.^25^ A concrete description of the control group in this study, however, is not provided. Most studies used the 16S rRNA sequencing technique for microbiome analysis. Lewis et al and Knoll et al performed metagenomic analyses in pediatric IBD patients and controls but lacked power due to limited sample sizes (86 IBD vs 26 HC, and 12 IBD vs 12 HC, respectively).^26^^,^^27^ Most studies on gut microbiome differences in pediatric IBD patients found similar results to ours, specifically a decreased alpha diversity in IBD patients compared to CGI and HCs and an increase in *E. coli* and *Bacteroides* species.^12^^,^^28–32^ This reduction in microbial diversity is a hallmark of gut dysbiosis, which is commonly associated with IBD.^30^ Beta diversity analysis revealed distinct clustering between groups, indicating significant compositional differences in the gut microbiome. This clustering supports the hypothesis that the gut microbiome in pediatric IBD patients is different from that of CGI and HC.

We identified 117 differentially abundant species between HC and IBD, 97 species between HC and CGI, and 32 species between CGI and IBD. An increase of *F. prausnitzii* and *E. coli* in IBD was observed when compared to HC, and a decrease of *A. communis* and an increase of *E. coli* in IBD when compared to CGI. *F. prausnitzii* is often featured in gut microbiome studies in IBD. Although our study found elevated levels of this bacterium in children with IBD, existing literature typically associates it with decreased abundance in IBD cases.^12^^,^^28^^,^^29^^,^^32^ It is known for its anti-inflammatory effects in the colon and is a major producer of butyrate, which also has anti-inflammatory properties.^33^ These characteristics are why *F. prausnitzii* is widely considered to have a protective role in the gut.^32^ A recent study reported lower *F. prausnitzii* levels in individuals at risk for CD prior to diagnosis.^34^ The increase in *F. prausnitzii* in our pediatric IBD group, however, appears contradictory to these findings. This discrepancy may be attributable to the inclusion of children with de novo IBD, reflecting a relatively early stage and shorter duration of disease. Interestingly, our subanalysis using the subspecies level revealed that the increased abundance of *F. prausnitizii SGB15316* was predictive for IBD, where the others SGBs of *F. prausnitzii* were not predictive. Adding subspecies-level analyses might reveal different associations compared to only species-level analyses, which could also explain why this increase in *F. prausnitzii* in IBD was not commonly observed before. These findings highlight the importance of subspecies-level resolution, as only 1 of the 8 detected *F. prausnitzii* subspecies (*SGB15316*) was predictive of IBD, suggesting functional heterogeneity within this species. Future comparative genomic analyses between this SGB and previously characterized protective strains may elucidate strain-specific roles in IBD pathogenesis. Our finding of a decreased abundance of *A. communis* is in accordance with the existing literature.^28^^,^^29^  *A. communis* belongs to the *Bacteroidales* order, which has frequently been associated with IBD.^35^ Similarly, the increased abundance of *E. coli* observed in our study is consistent with existing literature.^10^^,^^12^^,^^32^ Unlike obligate anaerobes such as *F. prausnitzii* and *A. communis*, *E. coli* is a facultative anaerobe that may have a competitive advantage in a higher oxidative stress environment.^36^

### Functional pathways

Functional analysis of the gut microbiome revealed an increased richness of metabolic pathways in IBD patients, suggesting an increased functional potential compared to both CGI and HC. It is important to note that the metabolic pathways of *E. coli* have been studied in much greater detail compared to those of other species.^37^ Consequently, many pathways in the MetaCyc database are linked to *E. coli*; therefore the increase in *E. coli* abundance observed in IBD patients likely contributes to the observed rise in pathway richness in IBD patients. Additionally, richness is a count of unique pathways and not directly tied to species diversity. However, this increase in functional potential may reflect the microbial community’s adaptive responses to the inflammatory conditions in the gut of IBD patients. Furthermore, stress-induced shifts in microbial gene expression or selection for metabolically versatile microbes may contribute to increased functional richness despite reduced microbial diversity.^10^ The distinct clustering of functional pathways and differential abundance findings further underscore the altered functional state of the gut microbiome in IBD. Notably, pathways involved in nucleotide degradation, particularly adenosine degradation, and palmitate biosynthesis were increased in IBD compared to both CGI and HC. Palmitates are the derivatives of palmitic acid, a fatty acid, and one study has suggested a potential link between palmitic acid and the onset of IBD.^38^ However, the precise role of this fatty acid in IBD pathogenesis remains to be further elucidated, as well as the significance of increased nucleotide degradation. It is important to highlight that while metagenomic data can be used to estimate pathway abundances, it does not indicate the actual levels of metabolites from these pathways. Therefore, future studies incorporating metabolomics are needed to clarify the role of these pathways in IBD.

### Diagnostic microbiome-based prediction of IBD

We created a microbiome-based diagnostic prediction model and found that the microbiome could effectively distinguish IBD from HC, with an AUC of 0.96 ± 0.02. The increased abundance of *E. coli* and the decreased abundance of *A. communis* were key microbial features driving the prediction of IBD. Adding clinical features or pathways to this model did not improve its accuracy. The predictive model distinguishing IBD from CGI showed an AUC of 0.71 ± 0.07 (species) and 0.61 ± 0.08 (pathways) when solely the microbiome data were used, an AUC of 0.90 ± 0.05 for the clinical model, and an AUC of 0.80 ± 0.06 when clinical features, species, and pathways were included in the model. This model was mainly driven by the clinical biomarkers CRP and FCP, increased abundance of *E. coli* and *A. faecicola*, and increased abundance of the 4-deoxy-L-threo-hex-4-enopyranuronate degradation pathway in IBD, and increased abundance of *SGB15272* in CGI. The strong performance of the clinical model in distinguishing IBD from CGI using CRP and FCP is expected, as these markers were also used to classify part of the CGI group, with low FCP levels being a key factor in this classification. Kim et al recently conducted fecal microbiome analyses through 16S rRNA sequencing in 785 IBD patients and 1462 HC and found an AUC of 0.99 for their diagnostic model based on the gut microbiome.^39^ This is comparable to our finding of an AUC of 0.96 for IBD vs HC. However, the distinction between IBD patients and CGI is a much more clinically relevant consideration for a diagnostic model.

In a post hoc analysis, we explored the potential of the gut microbiome to serve as a biomarker to prevent unnecessary endoscopies in CGI. Alpha diversity was lower (Figure S1B), and *E. coli* abundance was increased in the necessary endoscopy group compared to the unnecessary endoscopy group. We found that clinical features (AUC of 0.76 ± 0.11) similarly outperformed microbiome (AUC of 0.67 ± 0.11 for species, AUC of 0.71 ± 0.11 for pathways) in distinguishing the necessity of endoscopy, and combining both resulted in an AUC of 0.69 ± 0.10. Again, the superior performance of clinical features in this model is expected, since they determined whether a patient in this cohort would undergo an endoscopy. Consequently, our results do not convincingly show that the microbiome can replace or improve existing clinical decision tools. However, the small sample size of the unnecessary endoscopy group (*n* = 19) limits predictive power, as reflected in the large standard deviations. While clinical features showed the highest accuracy, clinical uncertainty still led to performing these endoscopies, which also holds value for reassurance for diagnosis despite negative endoscopy findings.

### Prediction of therapy response

We found no differences in baseline gut microbiome compositions between pediatric IBD patients who responded to induction therapy and those who did not, also when performing these analyses for the subgroup of patients receiving nutritional induction therapy. This aligns with some of the existing literature, which shows mixed findings on this topic, as only certain studies report a correlation between baseline microbiome diversity and future therapy response.^40^ Inconsistent results are also common regarding the predictive power of specific microbiome features in therapy response for IBD patients.^23^^,^^41^ For nutritional therapy specifically, a recent study reported 79% accuracy using microbiome features to differentiate between responders and nonresponders to EEN therapy.^18^ In our cohort, 17 out of 40 patients (42.5%) receiving nutritional therapy were classified as responders, while 23 patients (57.5%) were nonresponders. This nonresponse rate is higher than reported in the literature, which describes successful induction of remission in 75%–85% of cases with EEN and a 75% response rate to CDED at week 12.^42–45^ However, it is important to note that response definitions vary across studies, which may contribute to these inconsistent findings. While the microbiome might play a role in therapy response, the lack of predictive features in our study could be due to the complex and multifactorial nature of therapy response, as response is not only driven by the gut microbiome but also by immunological pathways, genetic factors, and disease severity and behavior.^46^ Further research with larger sample sizes is needed to elucidate the role of the gut microbiome in influencing therapy response.

### Limitations and strengths

First, due to the design of the study, where we collected a stool sample at one time point, we cannot draw conclusions on alterations in the gut microbiome during the course of the disease; this is a crucial step in understanding the role of the microbiome and its metabolites in the pathogenesis of IBD. Longitudinal studies with multiple sampling points and concurrent collection of clinical and biochemical disease activity data, along with mucosa-associated microbiome sampling, are needed. Such studies may also help clarify the cause-vs-consequence dilemma in microbiome research. Secondly, despite ours being one of the larger studies on the gut microbiome in pediatric IBD, the study population is still rather limited. Especially when divided into subgroups, sample size of IBD phenotypes and subtypes of CGI were fairly limited, complicating drawing conclusions on these subgroups. Moreover, data on diet and other environmental factors were limited, even though these are known influences on the gut microbiome.^47–49^ Additionally, not every individual in the CGI group underwent endoscopy, the reference standard for definitively ruling out IBD. However, we conducted a 1-year follow-up on all CGI patients after baseline and confirmed that none had developed IBD. Finally, while we did not perform follow-up endoscopies in the IBD group to assess mucosal healing for therapy response, we used standardized clinical scores to monitor outcomes.

While multiple studies, mainly in adults, have demonstrated that microbiome signatures can predict treatment response to biologics such as anti-TNF agents, vedolizumab, and ustekinumab, Prins et al reported only limited predictive value for ustekinumab and vedolizumab.^23^^,^^50^^,^^51^ In our study, we did not observe a strong microbiome-based predictive signal for treatment response, suggesting that its utility as a biomarker may not be universal. Factors such as disease severity, treatment history, and methodological differences across studies could have contributed to these discrepancies. Our pediatric cohort was characterized by relatively mild disease, with the majority being categorized as “mild” based on PCDAI and PUCAI, and minimal steroid and biologic use. Though these differences were not statistically significant, we acknowledge that disease severity at baseline may play a role in microbiome-treatment response associations.

An important strength of this study is the inclusion of a therapy-naïve cohort of de novo IBD patients. Previous microbiome studies in adult cohorts have often been limited by the influence of extensive therapy histories and the effects of long-term IBD.^12^^,^^36^ Some pharmacologic treatments are known to alter the microbiome, adding to heterogeneity in study populations.^52^^,^^53^ Similarly, most pediatric studies have focused on patients with established disease and treatment.^26–29^ An interesting future direction would be to compare our de novo therapy-naïve pediatric IBD group with established pediatric IBD and adult IBD. Moreover, by including not only HC but also CGI, our study closely mirrors clinical practice, helping to differentiate between new-onset IBD and non-IBD conditions. Though we found a statistically significant difference in age between IBD, HC, and CGI groups in this study population, we do not expect that this influenced outcome, as the composition and diversity of the intestinal microbiome reaches maturity and approaches that of an adult at the age of around 3 years.^54^^,^^55^ Furthermore, the use of shotgun metagenomic sequencing provides detailed subspecies-level taxonomic classifications and insight into microbial metabolic pathways, offering a more comprehensive understanding of the role of the microbiome in IBD pathogenesis than the commonly used 16S rRNA sequencing technique for microbiome analysis, especially for environments with high microbial densities such as the gut.^12^^,^^56^

## Conclusion

This study underscores the significant differences in gut microbiome diversity and composition between pediatric IBD patients, CGI, and HC. Our findings support the potential of the gut microbiome as a diagnostic tool for pediatric IBD, with an AUC of 0.96 for HC vs IBD and 0.71 for CGI vs IBD. Moreover, when combining clinical features with microbiome profiles and microbial metabolic pathways, AUCs of 0.96 (HC vs IBD) and 0.80 (CGI vs IBD) were achieved. Additionally, we highlight specific microbial and functional features that could serve as valuable biomarkers. While the baseline gut microbiome displayed limited predictive power for therapy response, it holds promise for enhancing diagnostic accuracy, and the predictive signature in microbiome features, mainly pathways, could potentially prevent unnecessary endoscopies. Further research is needed to validate these findings and explore the therapeutic potential of targeting the gut microbiome in IBD.

## Supplementary Material

izaf184_Supplementary_Data

## Data Availability

The data of the Rapid cohort are available upon reasonable request to the corresponding author. The sequencing data of the DMP cohort is available at the European Genome-Phenome Archive under accession EGAS00001005027 and can be accessed upon request.

## References

[1] Loftus EV. Jr ., Clinical epidemiology of inflammatory bowel disease: incidence, prevalence, and environmental influences. Gastroenterology. 2004;126:1504-1517. 10.1053/j.gastro.2004.01.063 15168363

[2] Benchimol EI , Fortinsky KJ, Gozdyra P, Van den Heuvel M, Van Limbergen J, Griffiths AM. Epidemiology of pediatric inflammatory bowel disease: a systematic review of international trends. Inflamm Bowel Dis. 2011;17:423-439. 10.1002/ibd.21349 20564651

[3] Roberts SE , Thorne K, Thapar N, et al A systematic review and meta-analysis of pediatric inflammatory bowel disease incidence and prevalence across europe. Journal of Crohn’s and Colitis. 2020;14:1119-1148. 10.1093/ecco-jcc/jjaa037 32115645

[4] Sawczenko A , Sandhu BK. Presenting features of inflammatory bowel disease in Great Britain and Ireland. Arch Dis Child. 2003;88:995-1000. 10.1136/adc.88.11.995 14612366 PMC1719349

[5] Henderson P , Anderson NH, Wilson DC. The diagnostic accuracy of fecal calprotectin during the investigation of suspected pediatric inflammatory bowel disease: a systematic review and meta-analysis. Am J Gastroenterol. 2014;109:637-645. 10.1038/ajg.2013.131 23670113

[6] Van Limbergen J , Russell RK, Drummond HE, et al Definition of phenotypic characteristics of childhood-onset inflammatory bowel disease. Gastroenterology. 2008;135:1114-1122. 10.1053/j.gastro.2008.06.081 18725221

[7] Heikenen JB , Werlin SL, Brown CW, Balint JP. Presenting symptoms and diagnostic lag in children with inflammatory bowel disease. Inflamm Bowel Dis. 1999;5:158-160. 10.1097/00054725-199908000-00002 10453370

[8] Levine A , Koletzko S, Turner D, et al; European Society of Pediatric Gastroenterology, Hepatology, and Nutrition. ESPGHAN revized porto criteria for the diagnosis of inflammatory bowel disease in children and adolescents. J Pediatr Gastroenterol Nutr. 2014;58:795-806. 10.1097/mpg.0000000000000239 24231644

[9] Chang JT. Pathophysiology of inflammatory bowel diseases. N Engl J Med. 2020;383:2652-2664. 10.1056/NEJMra2002697 33382932

[10] Lloyd-Price J , Arze C, Ananthakrishnan AN, et al; IBDMDB Investigators. Multi-omics of the gut microbial ecosystem in inflammatory bowel diseases. Nature. 2019;569:655-662. 10.1038/s41586-019-1237-9 31142855 PMC6650278

[11] Okou DT , Kugathasan S. Role of genetics in pediatric inflammatory bowel disease. Inflamm Bowel Dis. 2014;20:1878-1884. 10.1097/mib.0000000000000085 25118609 PMC4201539

[12] Ni J , Wu GD, Albenberg L, Tomov VT. Gut microbiota and IBD: causation or correlation? Nat Rev Gastroenterol Hepatol. 2017;14:573-584. 10.1038/nrgastro.2017.88 28743984 PMC5880536

[13] van Rheenen PF , Aloi M, Assa A, et al The medical management of pediatric Crohn’s disease: an ECCO-ESPGHAN guideline update. J Crohns Colitis. 2021;15:171–194. 10.1093/ecco-jcc/jjaa161 33026087

[14] Levine A , Sigall Boneh R, Wine E. Evolving role of diet in the pathogenesis and treatment of inflammatory bowel diseases. Gut. 2018;67:1726-1738. 10.1136/gutjnl-2017-315866 29777041

[15] Narula N , Dhillon A, Zhang D, Sherlock ME, Tondeur M, Zachos M. Enteral nutritional therapy for induction of remission in Crohn’s disease. Cochrane Database Syst Rev. 2018;4:Cd000542. 10.1002/14651858.CD000542.pub3 29607496 PMC6494406

[16] Yanai H , Levine A, Hirsch A, et al The Crohn’s disease exclusion diet for induction and maintenance of remission in adults with mild-to-moderate Crohn’s disease (CDED-AD): an open-label, pilot, randomized trial. Lancet Gastroenterol Hepatol. 2022;7:49-59. 10.1016/S2468-1253(21)00299-5 34739863

[17] Cozijnsen MA , van Pieterson M, Samsom JN, Escher JC, de Ridder L. Top-down infliximab study in kids with Crohn’s disease (TISKids): an international multicentre randomized controlled trial. BMJ Open Gastroenterol. 2016;3:e000123. 10.1136/bmjgast-2016-000123 PMC5223648 28090335

[18] Nichols B , Briola A, Logan M, et al Gut metabolome and microbiota signatures predict response to treatment with exclusive enteral nutrition in a prospective study in children with active Crohn’s disease. Am J Clin Nutr. 2024;119:885-895. 10.1016/j.ajcnut.2023.12.027 38569785 PMC11007740

[19] Höyhtyä M , Korpela K, Saqib S, et al Quantitative fecal microbiota profiles relate to therapy response during induction with tumor necrosis factor α antagonist infliximab in pediatric inflammatory bowel disease. Inflamm Bowel Dis. 2023;29:116-124. 10.1093/ibd/izac182 36040412 PMC9825283

[20] Hyams JS , Di Lorenzo C, Saps M, Shulman RJ, Staiano A, van Tilburg M. Functional disorders: children and adolescents. Gastroenterol. 2016;15:1456–1468. 10.1053/j.gastro.2016.02.015 27144632

[21] Gacesa R, Kurilshikov A, Vich Vila A, et al. Environmental factors shaping the gut microbiome in a Dutch population. Nature. 2022;604:732–739. 10.1038/s41586-022-04567-7 35418674

[22] Björk JR , Bolte LA, Maltez Thomas A, et al Longitudinal gut microbiome changes in immune checkpoint blockade-treated advanced melanoma. Nature Medicine. 2024;30:785–796. 2024/03/01. 10.1038/s41591-024-02803-3 PMC10957474 38365950

[23] Prins FM , Hidding IJ, Klaassen MAY, et al Limited predictive value of the gut microbiome and metabolome for response to biological therapy in inflammatory bowel disease. Gut Microbes. 2024;16:2391505. 10.1080/19490976.2024.2391505 39167702 PMC11340771

[24] Calle ML , Pujolassos M, Susin A. coda4microbiome: compositional data analysis for microbiome cross-sectional and longitudinal studies. BMC Bioinformatics. 2023;24:82. 2023/03/06. 10.1186/s12859-023-05205-3 36879227 PMC9990256

[25] Kellermayer R , Mir SA, Nagy-Szakal D, et al Microbiota separation and C-reactive protein elevation in treatment-naïve pediatric granulomatous Crohn disease. J Pediatr Gastroenterol Nutr. 2012;55:243-250. 10.1097/MPG.0b013e3182617c16 22699834 PMC3812911

[26] Lewis JD , Chen EZ, Baldassano RN, et al Inflammation, antibiotics, and diet as environmental stressors of the gut microbiome in pediatric Crohn’s disease. Cell Host Microbe. 2015;18:489-500. 10.1016/j.chom.2015.09.008 26468751 PMC4633303

[27] Knoll RL , Forslund K, Kultima JR, et al Gut microbiota differs between children with inflammatory bowel disease and healthy siblings in taxonomic and functional composition: a metagenomic analysis. Am J Physiol Gastrointest Liver Physiol. 2017;312:G327-g339. 10.1152/ajpgi.00293.2016 28039159

[28] Zhuang X , Liu C, Zhan S, et al Gut microbiota profile in pediatric patients with inflammatory bowel disease: a systematic review. Front Pediatr. 2021;9:626232. 10.3389/fped.2021.626232 33604319 PMC7884334

[29] Gevers D , Kugathasan S, Denson LA, et al The treatment-naive microbiome in new-onset Crohn’s disease. Cell Host Microbe. 2014;15:382-392. 10.1016/j.chom.2014.02.005 24629344 PMC4059512

[30] Olbjørn C , Cvancarova Småstuen M, Thiis-Evensen E, et al Fecal microbiota profiles in treatment-naïve pediatric inflammatory bowel disease—associations with disease phenotype, treatment, and outcome. Clin Exp Gastroenterol. 2019;12:37-49. 10.2147/ceg.S186235 30774408 PMC6362922

[31] Papa E , Docktor M, Smillie C, et al Non-invasive mapping of the gastrointestinal microbiota identifies children with inflammatory bowel disease. PLoS One. 2012;7:e39242. 10.1371/journal.pone.0039242 22768065 PMC3387146

[32] Pittayanon R , Lau JT, Leontiadis GI, et al Differences in gut microbiota in patients with vs without inflammatory bowel diseases: a systematic review. Gastroenterology. 2020;158:930-946.e1. 10.1053/j.gastro.2019.11.294 31812509

[33] Lopez-Siles M , Duncan SH, Garcia-Gil LJ, Martinez-Medina M. Fecalibacterium prausnitzii: from microbiology to diagnostics and prognostics. The ISME Journal. 2017;11:841-852. 2017/04/01. 10.1038/ismej.2016.176 28045459 PMC5364359

[34] Raygoza Garay JA , Turpin W, Lee S-H, et al; CCC GEM Project Research Consortium. Gut Microbiome composition is associated with future onset of Crohn’s disease in healthy first-degree relatives. Gastroenterology. 2023;165:670-681. 10.1053/j.gastro.2023.05.032 37263307

[35] Parker BJ , Wearsch PA, Veloo ACM, Rodriguez-Palacios A. The genus alistipes: gut bacteria with emerging implications to inflammation, cancer, and mental health. Front Immunol. 2020;11:906. 10.3389/fimmu.2020.00906 32582143 PMC7296073

[36] Morgan XC , Tickle TL, Sokol H, et al Dysfunction of the intestinal microbiome in inflammatory bowel disease and treatment. Genome Biol. 2012;13:R79. 10.1186/gb-2012-13-9-r79 23013615 PMC3506950

[37] Karp PD , Riley M, Saier M, Paulsen IT, Paley SM, Pellegrini-Toole A. The EcoCyc and MetaCyc databases. Nucleic Acids Res. 2000;28:56-59. 10.1093/nar/28.1.56 10592180 PMC102475

[388] Jezernik G , Potočnik U. Comprehensive genetic study of fatty acids helps explain the role of noncoding inflammatory bowel disease associated SNPs and fatty acid metabolism in disease pathogenesis. Prostaglandins Leukot Essent Fatty Acids. 2018;130:1-10. 10.1016/j.plefa.2018.02.002 29549916

[39] Kim H , Na JE, Kim S, et al A machine learning-based diagnostic model for Crohn’s disease and ulcerative colitis utilizing fecal microbiome analysis. Microorganisms. 2023;12:36. 38257863 10.3390/microorganisms12010036 PMC10820568

[40] Meade S , Liu Chen Kiow J, Massaro C, et al Gut microbiome-associated predictors as biomarkers of response to advanced therapies in inflammatory bowel disease: a systematic review. Gut Microbes. 2023;15:2287073. 10.1080/19490976.2023.2287073 38044504 PMC10730146

[41] Radhakrishnan ST , Alexander JL, Mullish BH, et al Systematic review: the association between the gut microbiota and medical therapies in inflammatory bowel disease. Aliment Pharmacol Ther. 2022;55:26-48. 10.1111/apt.16656 34751954

[42] Pigneur B , Lepage P, Mondot S, et al Mucosal healing and bacterial composition in response to enteral nutrition vs steroid-based induction therapy—a randomized prospective clinical trial in children with Crohn’s disease. J Crohn’s Colitis. 2019;13:846-855. 10.1093/ecco-jcc/jjy207 30541015

[43] Cohen-Dolev N , Sladek M, Hussey S, et al Differences in outcomes over time with exclusive enteral nutrition compared with steroids in children with mild to moderate Crohn’s disease: results from the GROWTH CD study. J Crohns Colitis. 2018;12:306-312. 10.1093/ecco-jcc/jjx150 29165666

[44] Spekhorst LM , Hummel TZ, Benninga MA, van Rheenen PF, Kindermann A. Adherence to oral maintenance treatment in adolescents with inflammatory bowel disease. J Pediatr Gastroenterol Nutr. 2016;62:264-270. 10.1097/mpg.0000000000000924 26230905

[45] Levine A , Wine E, Assa A, et al Crohn’s disease exclusion diet plus partial enteral nutrition induces sustained remission in a randomized controlled trial. Gastroenterol. 2019;157:440-450.e8. 10.1053/j.gastro.2019.04.021 31170412

[46] Elhag DA , Kumar M, Saadaoui M, et al Inflammatory bowel disease treatments and predictive biomarkers of therapeutic response. Int J Mol Sci. 2022;23: 10.3390/ijms23136966 PMC9266456 35805965

[47] David LA , Maurice CF, Carmody RN, et al Diet rapidly and reproducibly alters the human gut microbiome. Nature. 2014;505:559-563. 10.1038/nature12820 24336217 PMC3957428

[48] Brown K , DeCoffe D, Molcan E, Gibson DL. Diet-induced dysbiosis of the intestinal microbiota and the effects on immunity and disease. Nutrients. 2012;4:1095-1119. 10.3390/nu4081095 23016134 PMC3448089

[49] Zhang YZ , Li YY. Inflammatory bowel disease: pathogenesis. World J Gastroenterol. 2014;20:91-99. 10.3748/wjg.v20.i1.91 24415861 PMC3886036

[50] Ananthakrishnan AN , Luo C, Yajnik V, et al Gut microbiome function predicts response to anti-integrin biologic therapy in inflammatory bowel diseases. Cell Host Microbe. 2017;21:603-610.e3. 10.1016/j.chom.2017.04.010 28494241 PMC5705050

[51] Caenepeel C , Falony G, Machiels K, et al Dysbiosis and associated stool features improve prediction of response to biological therapy in inflammatory bowel disease. Gastroenterol. 2024;166:483-495. 10.1053/j.gastro.2023.11.304 38096956

[52] Wada H , Miyoshi J, Kuronuma S, et al 5-Aminosalicylic acid alters the gut microbiota and altered microbiota transmitted vertically to offspring have protective effects against colitis. Scientific Reports. 2023;13:12241. 2023/07/28. 10.1038/s41598-023-39491-x 37507482 PMC10382598

[53] O'Reilly C , Mills S, Rea MC, et al Interplay between inflammatory bowel disease therapeutics and the gut microbiome reveals opportunities for novel treatment approaches. Microbiome Res Rep. 2023;2:35. 10.20517/mrr.2023.41 37849974 PMC7615213

[54] Rinninella E , Raoul P, Cintoni M, et al What is the healthy gut microbiota composition? A changing ecosystem across age, environment, diet, and diseases. Microorganisms. 2019;7. 10.3390/microorganisms7010014 PMC6351938 30634578

[55] Yatsunenko T , Rey FE, Manary MJ, et al Human gut microbiome viewed across age and geography. Nature. 2012;486:222-227. 10.1038/nature11053 22699611 PMC3376388

[56] Collij V , Klaassen MAY, Weersma RK, Vila AV. Gut microbiota in inflammatory bowel diseases: moving from basic science to clinical applications. Hum Genet. 2021;140:703-708. 10.1007/s00439-020-02218-3 32857194 PMC8052217

