# Supplementary material for: Metagenomic Sequencing Reveals Distinct Gut Microbiome Profiles in Therapy-Naïve de Novo Pediatric Inflammatory Bowel Disease
Source: Inflamm Bowel Dis. 2025 Sep 12;32(2):207–19. doi: 10.1093/ibd/izaf184 (PMC12857426; doi:10.1093/ibd/izaf184)
Supplement: izaf184_Supplementary_Data [file izaf184_supplementary_data.docx]

# Supplementary Material

**Supplementary Table S1: Final diagnoses of the controls with gastrointestinal symptoms.**

Abbreviations: IBS, irritable bowel syndrome; FAP-NOS, functional abdominal pain-not otherwise specified; IBD, inflammatory bowel disease.

|  | **Patients**, n (%) |
| --- | --- |
|  |  |
| IBS | 34 (45.3) |
| FAP-NOS | 18 (24.0) |
| Polyp | 6 (8.0) |
| Post-infectious | 5 (6.7) |
| No alternative diagnosis (transient symptoms) | 3 (4.0) |
| Non-specific colitis (non-IBD) | 2 (2.7) |
| Abdominal migraine | 1 (1.3) |
| Chronic appendicitis | 1 (1.3) |
| Coeliac disease | 1 (1.3) |
| Gynaecological disease | 1 (1.3) |
| Helicobacter pylori gastritis | 1 (1.3) |
| Protein-losing enteropathy | 1 (1.3) |
| Ulcer seam (after prior intestinal resection) | 1 (1.3) |

**Supplementary Table S2:** **Characteristics of the IBD cases**.

Abbreviations: CRP, C-reactive protein; IBD; inflammatory bowel disease; PCDAI, Paediatric Crohn’s Disease Activity Index; PUCAI; Paediatric Ulcerative Colitis Activity Index; 5-ASA, 5-aminosalicylic acid; anti-TNF, anti-tumour necrosis factor; CDED, Crohn’s disease exclusion diet; EEN, exclusive enteral nutrition; PEN, partial enteral nutrition; MTX, methotrexate.

|  | **Crohn's disease (CD)** | **Ulcerative colitis (UC)** | **IBD-unclassified (IBD-U)** |  |
| --- | --- | --- | --- | --- |
|  |  |  |  | p-value^5^ |
|  | n = 70 | n = 27 | n =6 |  |
| Female | 36 (51%) | 15 (56%) | 3 (50%) | 0.943 |
| Age, years | 15 (13-16) | 14 (12.5-15) | 13.5 (12.25-14.75) | 0.158 |
| Body mass index^1^, kg/m^2^ | 18.9 (15.9-21.1) | 18.5 (17.2-21.5) | 18.7 (17.3-21.0) | 0.910 |
| Faecal calprotectin^1^, µg/kg | 1807 (886-2987) | 2171 (1401-4137) | 2561 (1545-3334) | 0.377 |
| CRP^1^, mg/L | 12 (6-47) | 1 (1-8) | 1 (1-1) | **<0.001** |
| Reads, million | 12.6 (10.7-14.1) | 8.4 (5.3-12.3) | 11.4 (9.1-14.2)) | **0.002** |
| **IBD characteristics** |  |  |  |  |
| PCDAI/ PUCAI (baseline) | 30 (23-42) | 45 (30-49) | 30 (26-34) | **0.011** |
| Disease severity^1,2^ |  |  |  | 0.411 |
| *mild* | 31 (47%) | 9 (35%) | 4 (67%) |  |
| *moderate* | 16 (24%) | 11 (42%) | 2 (33%) |  |
| *severe* | 18 (27%) | 6 (23%) | 0 (0%) |  |
| Induction therapy^3^ |  |  |  | **<0.001** |
| *5-ASA* | 0 (0%) | 23 (85%) | 5 (83%) |  |
| *Anti-TNF alpha agent* | 5 (7.1%) | 0 (0%) | 0 (0%) |  |
| *Steroids* | 1 (1.4%) | 4 (15%) | 0 (0%) |  |
| *Nutritional therapy* | 56 (80%) | 0 (0%) | 0 (0%) |  |
| *Combination of therapies* | 8 (11%) | 0 (0%) | 1 (17%) |  |
| Response to therapy^4^ |  |  |  | 0.932 |
| *Responders* | 18 (43%) | 9 (47%) | 3 (50%) |  |
| *Non-responders* | 24 (57%) | 10 (53%) | 3 (50%) |  |
| *Unknown* | 28 | 8 | 0 |  |

^1^ Missing values for one or more of the groups

^2^ One individual in the CD group was classified as in remission based on the clinical score at baseline

^3^ Anti-TNF alpha agents included infliximab and adalimumab. Nutritional therapy included CDED, EEN or CDED + PEN. Combination of therapies included 5-ASA + nutritional therapy, metronidazole + nutritional therapy, MTX + nutritional therapy, anti-TNF + ciprofloxacin or thiopurine or MTX, and azithromycin/metronidazole + nutritional therapy

^4^ Response to therapy defined as both clinical response (reduction in clinical scores) and absence of the need for treatment escalation.

^5^ Pearson’s Chi-squared test for categorical variables, Kruskal-Wallis test for numerical variables.

**Supplementary Table S3:** **Characteristics of responder versus non-responders**.

Abbreviations: CRP, C-reactive protein; IBD; inflammatory bowel disease; PCDAI, Paediatric Crohn’s Disease Activity Index; PUCAI; Paediatric Ulcerative Colitis Activity Index; 5-ASA, 5-aminosalicylic acid; anti-TNF, anti-tumour necrosis factor; CDED, Crohn’s disease exclusion diet; EEN, exclusive enteral nutrition; PEN, partial enteral nutrition; MTX, methotrexate.

|  | **Responders** | **Non-responders** |  |
| --- | --- | --- | --- |
|  |  |  | p-value^4^ |
|  | n = 30 | n = 37 |  |
| Female | 18 (60%) | 19 (51%) | 0.479 |
| Age, years | 15(12-16) | 15 (13-16) | 0.969 |
| Body mass index^1^, kg/m^2^ | 18.2(16.6-21.2) | 18.6 (15.9-21.3) | 0.913 |
| Faecal calprotectin^1^, µg/kg | 1810 (1459-3000) | 2050 (990-3250) | 0.882 |
| CRP^1^, mg/L | 8 (2-34) | 5 (1-12) | 0.176 |
| Reads, million | 11.9 (8.6-13.2) | 11.4 (9.0-12.9) | 0.702 |
| **IBD characteristics** |  |  |  |
| PCDAI/ PUCAI (baseline) | 30 (28-44) | 28 (20-40) | 0.170 |
| PCDAI/ PUCAI (3 months) | 0 (0-5) | 10 (5-20) | **<0.001** |
| IBD type |  |  | 0.932 |
| *Crohn’s disease* | 18 (60%) | 24 (65%) |  |
| *Ulcerative colitis* | 9 (30%0 | 10 (27%) |  |
| *IBD-unclassified* | 3 (10%) | 3 (8.1%) |  |
| Disease severity^1,2^ |  |  | 0.538 |
| *mild* | 14 (47%) | 20 (54%)) |  |
| *moderate* | 12 (40%) | 10 (27%) |  |
| *severe* | 4 (13%) | 7 (19%) |  |
| Induction therapy^3^ |  |  | 0.838 |
| *5-ASA* | 12 (40%) | 12 (32%) |  |
| *Nutritional therapy* | 17 (57%) | 23 (62%) |  |
| *Combination of therapies* | 1 (3.3%) | 2 (5.4%) |  |

^1^ Missing values for one or more of the groups

^2^ One individual in the CD group was classified as in remission based on the clinical score at baseline

^3^ Nutritional therapy included CDED, EEN or CDED + PEN. Combination of therapies included 5-ASA + nutritional therapy, metronidazole + nutritional therapy, MTX + nutritional therapy, anti-TNF + ciprofloxacin or thiopurine or MTX, and azithromycin/metronidazole + nutritional therapy

^4^ Pearson’s Chi-sqared test for categorical variables, Mann-Witney U test for numerical variables.


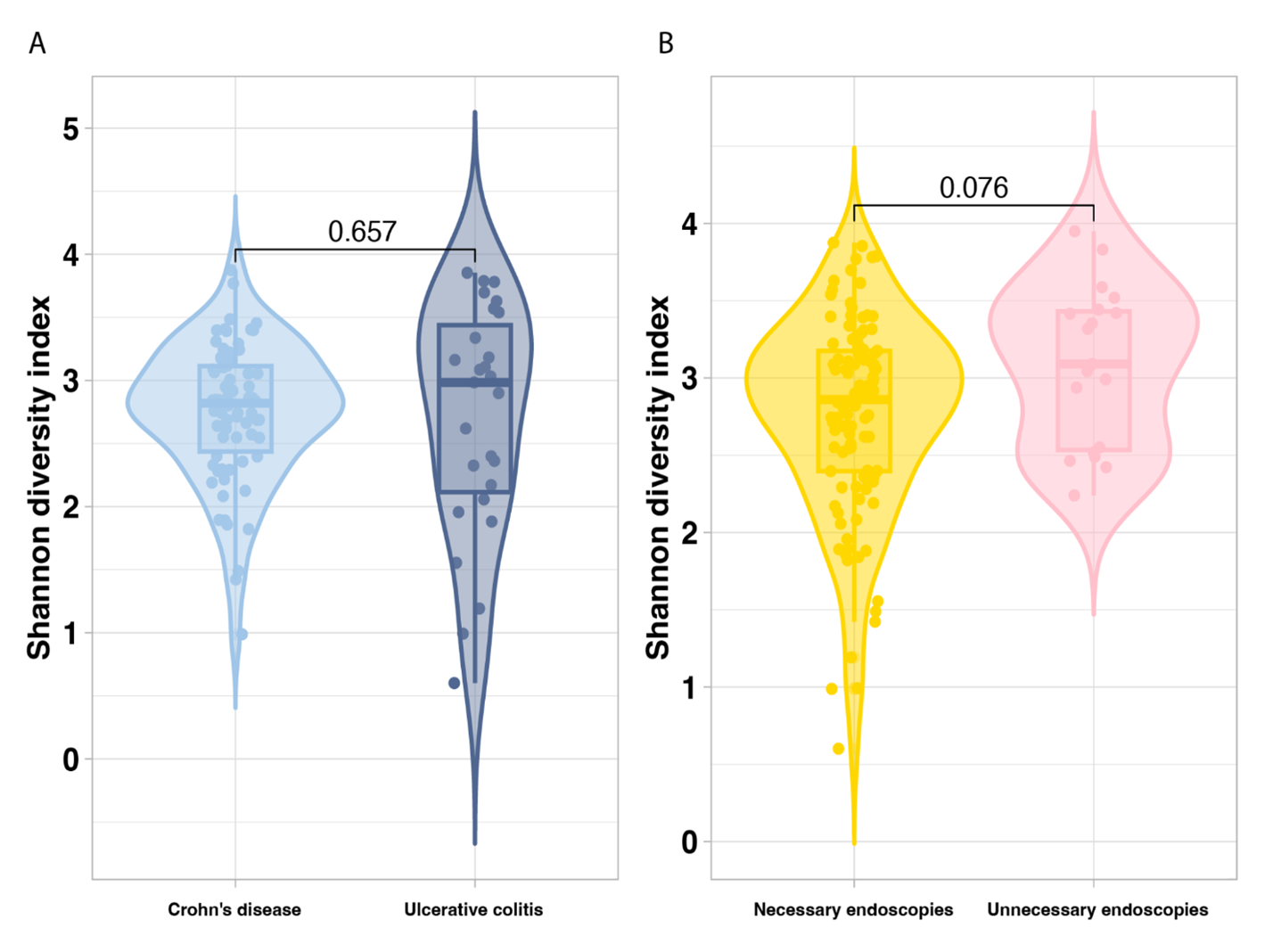
**Supplementary Figure S1.** Shannon diversity in supplemental analysis
**A)** showing the alpha diversity determined using the Shannon diversity index for CD and UC. **B)** The alpha diversity between the necessary endoscopy and the unnecessary endoscopy groups. No significant differences were observed.

Abbreviations: CD, Crohn’s disease; UC, ulcerative colitis.


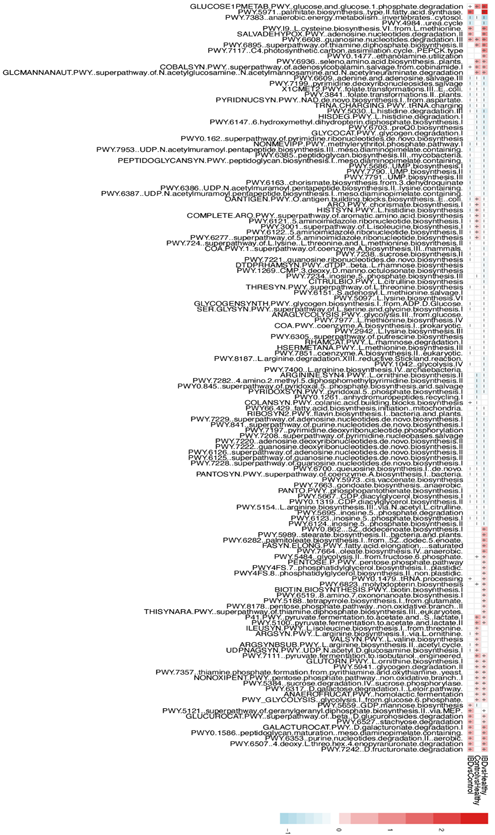


**Supplementary Figure S2:** Differential abundance pathway analysis. Shown are the pathways that were significantly different between the comparison listed: IBD vs HC, CGI vs HC and IBD vs CGI.

Abbreviations: IBD, inflammatory bowel disease; HC, healthy control; CGI, controls with gastrointestinal symptoms.
